# Electrochemical analysis of anticancer and antibiotic drugs in water and biological specimens

**DOI:** 10.1039/d4ra05685j

**Published:** 2024-11-18

**Authors:** Ayesha Qureshi, Afzal Shah, Faiza Jan Iftikhar, Abdul Haleem, Muhammad Abid Zia

**Affiliations:** a Department of Chemistry, Quaid-i-Azam University Islamabad 45320 Pakistan afzals_qau@yahoo.com; b National University of Technology (NUTECH) Islamabad 44000 Pakistan; c Department of Chemistry, University of Education Attock Punjab 43600 Pakistan

## Abstract

The increasing prevalence of pharmaceuticals in water and complex matrices necessitates accurate measurement and monitoring of their environmental contamination levels. This is crucial not only for environmental conservation but also for comprehending the intricate mechanisms involved and developing more effective treatment approaches. In this context, electrochemical techniques show significant potential for the detection of pharmaceuticals across various matrices. Specifically, voltammetry is advantageous due to its rapid, straightforward, and cost-effective nature, allowing for the simultaneous analysis of multiple anticancer and antibiotic drugs. By utilizing nanomaterial-modified electrochemical sensors, the sensitivity and selectivity of detection methods can be significantly improved. The small size and customizable properties of nanomaterials enable these sensors to identify trace amounts of drugs in diverse samples. However, challenges persist in achieving reliable and accurate electrochemical monitoring of drugs in water and biological samples. Biofluids such as saliva, urine, and blood/serum, along with environmental samples from lakes and rivers, often contain numerous interfering substances that can diminish analyte signals. This review examines electrochemical methods and their potential applications for detecting pharmaceuticals and their metabolites, while also addressing the mechanisms of action and harmful effects of these drugs on both ecosystems and human health. Recent developments in electrochemical sensors utilizing nanomaterials for the detection of health-threatening pharmaceutical contaminants are examined, providing important insights into their underlying mechanisms. The emphasis is placed on the detection of anticancer agents and antibiotics, which relies on the electrocatalytic properties of the sensor materials. Additionally, discussions on density functional theory studies are included, along with an exploration of the emerging challenges and future directions in this area, aimed at enhancing readers' comprehension of the field and underscoring the necessary actions for a sustainable future.

## Abstract 2

The increasing prevalence of pharmaceuticals in water and complex matrices necessitates accurate measurement and monitoring of their environmental contamination levels.

## Graphical abstract

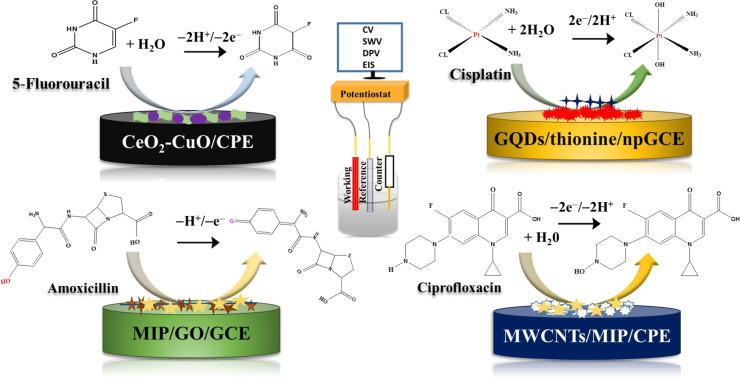

## 1. Introduction

**Fig. 1:**
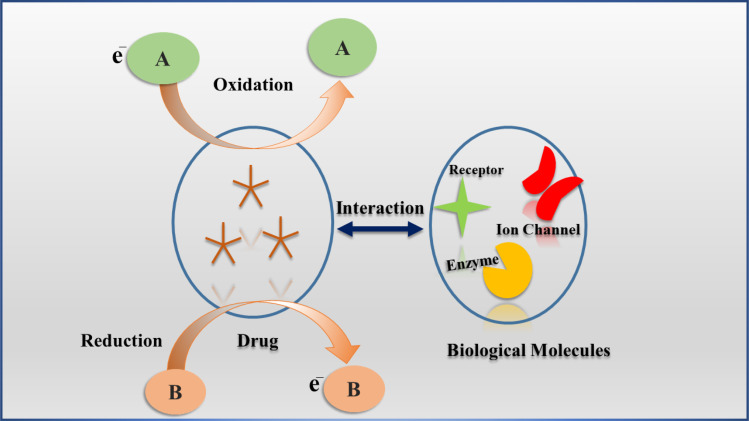
Schematic representation of the electrochemistry of a drug inside the body.

Drugs play a crucial role in human health, serving both medicinal and recreational functions. For their therapeutic effects, drugs engage with specific biomolecules in the body through redox reactions, which are vital to their mechanisms of action.^1^ Consequently, numerous drugs experience oxidation or reduction processes that either activate or deactivate their biological effects. The redox properties of electroactive drugs allow researchers to explore the electron transfer dynamics between these drugs and biomolecules.^2^Fig. 1 depicts the interaction between an electroactive drug and biological molecules, highlighting its electrochemical behavior within the body. These drugs possess electroactive properties, and the figure demonstrates their operational mechanism through redox processes that facilitate interactions with biological molecules. During this redox reaction, one molecule undergoes oxidation by losing an electron, while another molecule is reduced by gaining an electron, thereby enabling electron transfer, which is essential for comprehending the drug's interaction mechanism with biomolecules.

Numerous studies have been conducted to explain the action mechanism of anticancer and antibiotic drugs but still, the detailed mechanism is not fully understood. Therefore, electrochemical analysis of these drugs is performed in different matrices like urine, sweat, saliva, serum, foodstuff, and water samples to get insights into their redox fate. This approach could help understand whether an electron transfer or proton-coupled electron transfer is involved so that the action mechanism of anticancer and bactericidal role is inferred and elucidated upon.^3,4^ The mechanistic information is expected to help specify drug dosage to reduce toxicity and cost as tons of anticancer medicines are used annually in chemotherapy while antibiotics are also being widely used in human and animal medications. Despite their benefits to living organisms, they have been reported to be hazardous at all levels of the biological hierarchy.^5,6^ In the light of these facts, many environmental researchers have examined the adverse effects of these medications on animals, humans, and other organisms and attempted to offer removal techniques.^7^

Anticancer and bactericidal drugs are typically administrated at low concentrations. After being administered into the body, they undergo digestion and form metabolites in trace amounts. Monitoring them has proven to be extremely difficult due to significant interference of complex biological and environmental sample matrix, the ability to accumulate in food chains, and the spread of antibiotic-resistant bacteria.^8,9^ Hence, to understand their profile, several methods of analysis notably titrimetry, spectrophotometry, liquid chromatography, and voltammetry have been reported for determining the concentration of these medications and their metabolites. Liquid chromatography is a time-consuming technique, while titrimetry and spectrophotometry necessitate an extraction phase prior to analysis, indicating a demand for a more efficient alternative for routine testing. Electrochemical methods present practical solutions, as they are straightforward and rapid, eliminating the need for any supplementary steps to assess the analytes.^10^

Until now, the most commonly used voltammetric techniques for monitoring anticancer and antibiotic drugs are cyclic voltammetry (CV), linear sweep voltammetry (LSV), differential pulse voltammetry (DPV), and square wave voltammetry (SWV).^11^ It has been noted that relying solely on a single voltammetric technique may not suffice for the reliable and accurate detection of pharmaceuticals and their residues due to inherent limitations. To overcome this challenge, a combination of various techniques, such as anodic stripping voltammetry (ASV) and adsorptive stripping voltammetry (AdSV), along with square wave voltammetry (SWV) and differential pulse voltammetry (DPV), including methods like differential pulse anodic stripping voltammetry (DPASV), adsorptive differential pulse voltammetry (AdSDPV), adsorptive square wave voltammetry (AdSWV), and square wave adsorptive voltammetry (SWAdSV), has been employed due to their enhanced sensitivity compared to traditional methods for trace analysis.^12^ The high resolution provided by DPV and SWV, when combined with the sensitivity of stripping voltammetry, not only results in a superior signal-to-noise ratio but also enables the trace detection of pharmaceuticals in the presence of interferents within complex matrices. In ASV, heavy metal ions are pre-concentrated on the electrode surface, followed by quantification through anodic potential sweeping, while AdSV enhances sensitivity and selectivity for the target analyte by adsorbing the electroactive compound onto the electrode surface.^13^ The key features of these methods include simplicity of analysis, selectivity at specific formal potentials which can allow multiple analyte monitoring without the need for separation steps, and the skill to investigate biological samples such as urine, serum, and sweat. In addition to having comparatively quick sample times, amplification of mass transport, and affordability, voltammetric approaches can provide detailed information about how a drug will be consumed within the body at specific dosage levels and enable studies of how pharmaceuticals interact with living cells.^14,15^

Electrochemical sensors utilizing nanomaterials have attracted considerable attention over recent decades. The progress in the synthesis of nanomaterials has facilitated the creation of tunable morphologies, dimensions, surface charges, and physiochemical properties, which are crucial for achieving rapid, specific, and reliable detection of various molecules.^16^ These nanomaterials enhance the performance of modified electrodes by providing a larger surface area, resulting in an increased number of active sites that improve sensitivity. Furthermore, the functionalization of nanomaterials enhances selectivity by focusing on specific molecules while minimizing interference. They also enable lower detection limits for target analytes within complex matrices. The distinctive characteristics of nanomaterials have significantly enhanced the analytical capabilities of electrochemical sensors.^17^ However, to fully exploit these sensors, it is vital to have a comprehensive understanding of the physiochemical and electronic interactions occurring at the interface between the drug and the nanomaterial-based electrode.^18^

It is widely acknowledged that sensor materials are essential to the establishment of an electrochemical sensing platform capable of differentiating target molecules using various electrochemical analytical techniques.^19^ This document outlines various nanomaterial-based sensors that have been extensively researched and employed for the rapid detection and measurement of anticancer and antibiotic medications. The study focuses on the advancement of various electrochemical methods, particularly voltammetry, emphasizing their sensitivity, efficiency, and rapid analysis for identifying pharmaceuticals in environmental and biological matrices. It investigates the role of nano-based sensors in monitoring drug residues within biological samples for pharmacokinetic studies and therapeutic drug monitoring, as well as in water samples where these substances and their metabolites threaten ecological balance. Additionally, the research examines electrochemical sensors utilizing nanomaterials, analyzing their responses through oxidation or reduction mechanisms for the detection of anticancer and antibiotic drugs. Lastly, it provides a critical evaluation of electrochemical techniques, addressing the challenges encountered in trace-level detection and offering perspectives for future developments in this research domain.

## 2. Anticancer and antibiotic drugs

Cancer is a worldwide life-threatening issue and its treatment process is intricate involving diagnosis, therapy, monitoring, and prevention.^20^ Despite numerous advantages of anticancer drugs, these medications pose serious health issues such as anemia, appetite loss, constipation, fatigue, *etc.* as well as high potential for toxicity and mutagenicity.^21^ In parallel, antibiotics have become groundbreaking treatments for many infectious diseases. However, if leftover antibiotics are present in excess in the body, they might be dangerous since they can lead to infections like gonorrhea, pneumonia, tuberculosis as well as antibiotic resistance and allergic reactions in humans.^22^ A lot of drugs fall into the category of anticancer and antibiotics but in this review, we discuss the most frequently used toxic drugs from both categories such as tamoxifen, etoposide, 5-fluorouracil, doxorubicin, imatinib, mebendazole, sulphadiazine, metronidazole, levofloxacin, chloramphenicol, ofloxacin and ciprofloxacin. In addition to the aforementioned medications, platinum-based drugs, including cisplatin, carboplatin, and oxaliplatin, as well as β-lactams such as cefixime and amoxicillin, are frequently utilized as anticancer and antibiotic agents, respectively. Table 1 illustrates some of the typical applications and potential adverse effects associated with these anticancer and antibiotic drugs.

**Table 1:** Anticancer and antibiotics drug usage and their associated toxic effects

Drug type	S. No	Name of drugs	Uses	Harmful effects	Ref.
	1	Tamoxifen	Selective modulator of estrogen receptors, treatment of breast cancer	Mood swings	23
				Dry skin	
				Vision problems	
	2	Etoposide	Treatment of various malignancies such as lung, stomach, testicles, and bladder	Constipation	24
				Fatigue	
				Sore throat	
	3	5-Fluorouracil	Treatment of solid tumors such as (neck, stomach, head, breast, liver, and pancreatic cancer)	Diarrhea	25
				Vomiting	
				Nausea	
	4	Doxorubicin	First-line cure for breast cancer	Cardiotoxicity	26
				Alopecia	
	5	Imatinib	Anticancer	Low plasma concentration	27
	6	Mebendazole	Cure infections linked to whipworm, ascaris, and hookworms	Loss of appetite	28
				Abdominal pain	
	7	Cisplatin	Treat tumor cells	Hair loss, hypersensitivity, vomiting, mouth sores	29
	8	Carboplatin	Treat ovarian cancer, head and neck cancer	Nephrotoxicity, myelodepression	30
	9	Oxaliplatin	Cure tumor cells	Accumulation in body tissues	31
Antibiotic	10	Sulphadiazine	Utilized in aquaculture and human healthcare	Allergic & toxic reactions in the body	32
	11	Metronidazole		Ataxia	33
				Seizures	
				Neuropathy	
	12	Levofloxacin	Treat respiratory, cutaneous, urinary tract, and soft tissues infections	Tendinopathy	34
				Muscle atrophy	
	13	Chloramphenicol	Treat infectious disorders of both animals and humans	Gray baby syndrome	35
				Leukemia	
	14	Ofloxacin	Treat respiratory infections such as (pneumonia, and bronchitis), infections of skin and reproductive organs	Tendon damage	36
				Peripheral neuropathy	
	15	Ciprofloxacin	Used against infections including urinary tract infection (UTI), chronic bacterium prostatitis, and respiratory tract	Nerve damage, vomiting	37
	16	Cefixime	Used to cure infections like UTI, bronchitis, gonorrhea pneumonia, *etc.*	Bleeding or bruising	38
	17	Amoxicillin	Treat pneumonia, UTI, and middle ear infections	Nausea, rashes, vomiting	39

The detrimental impact of these drugs on human health and other organisms underscores the necessity for highly sensitive analytical instruments to identify and track trace levels of pharmaceutical residues. Even small amounts of certain anticancer and antibiotic medications can adversely affect ecosystems, disrupting aquatic life and jeopardizing human health, thereby influencing the overall quality of our food and water supplies. Furthermore, traditional methods for identifying and monitoring environmental pollutants present significant challenges for researchers, as these substances often exist in trace concentrations and are frequently accompanied by various interfering agents that complicate their detection. Given that the environment is dynamic, with contaminants capable of dispersing, transforming, and degrading over time, there is a pressing need for real-time or near-real-time monitoring capabilities to provide timely insights and enable swift responses to emerging threats. To effectively assess a broad spectrum of environmental toxins, it is essential to utilize a detection technique that is both adaptable and multifunctional, capable of identifying a diverse range of contaminants, including heavy metals, organic chemicals, dyes, pharmaceuticals, and novel toxins.

In order to make routine monitoring of environmental pollutants feasible and accessible, analytical methods and detection techniques must be made affordable. However, challenges such as costly techniques in resource-limited settings, the variability and complexity of pollutants, the necessity to enhance the sensitivity of the monitoring technique and the need to meet the regulatory compliance for environmental pollutants by updating and validating the techniques, need to be addressed. Hence these challenges necessitate the design and development of advanced, powerful and cost effective analytical methods and detection devices.

## 3. Detection techniques

**Scheme 1:**
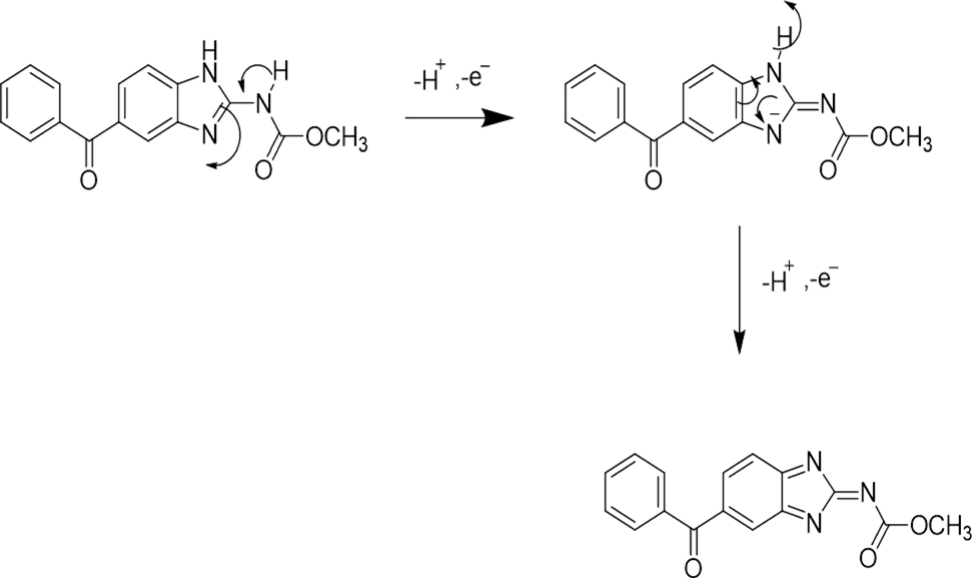
Proposed oxidation mechanism of the drug mebendazole at POA/MWCNT/GCE.^48^

Various detection techniques and methods of analysis such as spectrophotometry,^40^ high-performance liquid chromatography (HPLC),^41^ gas chromatography (GC),^42^ and mass spectrometry (MS)^43^ have been reported to detect environmental pollutants. Spectrophotometry is simple but poses limitations in terms of detection accuracy while other monitoring techniques such as HPLC, GC, and MS offer good detection accuracy, but their operation is complex due to the requirement of precise calibration procedures, bulky equipment and specialized personal to handle the instrument, and necessitate more effective alternate methods for real time and on-site detection.^44^ In this regard electrochemical techniques such as CV, LSV, DPV, SWV *etc.* are preferred by researchers for the detection of different analytes.^45^ These offer excellent sensitivity, affordability, accuracy, and less expensive instrumentation.^46^ All of these approaches have their own strengths. For example, CV presents a comprehensive examination of both oxidation and reduction processes in a single experiment while SWV, LSV, and DPV typically are focused on obtaining results in a single direction of potential scanning.^47^ For instance, Scheme 1 represents the oxidation mechanism of the drug mebendazole (MBZ) at glassy carbon electrode (GCE) modified by multiwalled carbon nanotubes (MWCNT) and poly-(*o*-anisidine) (POA) nanocomposite material.^48^ The provided mechanism indicates that MBZ experiences electrooxidation through the transfer of two electrons and two protons associated with the benzimidazole structure. The presence of POA, which contains an electron-donating methoxy group, enhances the electron density of the molecule, thereby influencing the oxidation process of MBZ. Additionally, the transfer of hydrogen from MBZ to POA contributes to the stabilization of the intermediates generated during the oxidation of MBZ at a designated potential. Thus, voltammetric techniques help figure out redox reduction potentials, thermodynamic parameters, and kinetics of the electrochemical processes by employing electrochemical sensors.

## 4. Electrochemical sensors

In recent years, electrochemical sensors have received a lot of attention for determining analytes in diverse media. Among the numerous types of analytical procedures and devices, electrochemical sensors are being pursued as they offer high selectivity, sensitivity, and reliability for detection of analytes. These electrochemical sensors have tailored sensing platforms that are characterized by various materials to identify specific target analytes.^49,50^ In specific circumstances, the performance of electrodes is augmented through the incorporation of nanomaterials, which enhances their selectivity, stability, and sensitivity towards the target analyte.^51^ The emergence of nanomaterials and the modification of electrodes have significantly influenced the advancement of electrochemical sensors in research. Due to their unique properties, including a high surface area for adsorption and distinct electrical, magnetic, and optical features relative to bulk materials, nanomaterials are commonly employed, along with their ease of modification.^52^ Additionally, nanomaterials are in the nano-range scale which aids in the miniaturization of electrochemical appliances. It is worth mentioning that electrochemical sensors can be made inexpensive, handy, quick and accurate for various analytes, provided the electrode is skilfully modified and built.

The use of various modifiers, including carbon-based nanomaterials like carbon nanotubes, graphene, and quantum dots, as well as metallic-based nanomaterials such as transition metals and metal oxides, polymer-based nanomaterials like poly(glycine), poly(pyrrole), and polyaniline, along with hybrid nanomaterials and molecularly imprinted polymers, is crucial for the detection of target analytes. These materials enhance the electrochemical surface area and offer exceptional conductivity, thereby improving detection capabilities.^53,54^ In carbon-based nanomaterials the use of graphene and its derived materials, carbon nanotubes (SWCNTs, MWCNTs), and carbon nanofillers (metallic organic framework MOFs) are most commonly employed in comparison to others. They exhibit high sensitivity, fast electron transfer, low detection limits and wide potential windows in aqueous environment.^55^

Metallic nanomaterials enhance the sensitivity of modified electrodes by improving electron transfer and mass transport rates. Furthermore, their high proportion of surface atoms with unoccupied valences contributes to the increased activity of various chemical processes.^56,57^ Recent research has identified nanopolymers as highly adaptable nanostructures utilized in various shapes and configurations. These nanopolymers demonstrate unique electrochemical properties due to nanoscale interactions between the polymers and their surroundings, characteristics that are absent in their bulk counterparts.^58^ Hybrid nanomaterials have garnered significant interest in research due to their ability to serve as adaptable frameworks for developing innovative nanomaterials. This is attributed to their high surface-to-volume ratio and the diminutive size of their structural components, which contribute to their distinctive properties.^59^ Molecularly imprinted polymers have garnered considerable interest due to their exceptional selectivity, cost-effectiveness, ease of use, compactness, and capacity to interact with specific target molecules across various matrices for drug analysis. They can be seamlessly integrated with chemical sensors or utilized alongside diverse analytical instruments, offering insights into analytes such as biological samples and drug dosage.^60^ The detection mechanism significantly contrasts with direct electrochemical methods, which rely on redox reactions occurring at the surface of nano-based electrodes for pharmaceutical detection. In contrast, electrochemical sensors based on MIPs utilize a recognition step that allows target molecules to attach to imprinted sites on synthetic polymers fixed to an electrode, with these sites being complementary in shape and size to the target molecules. This mimicking of biological receptors enables the differentiation of drug molecules even within complex matrices, thereby reducing interferences and proving particularly advantageous for clinical diagnostics that demand accurate drug detection. Moreover, the applications of biosensors have also been documented in the literature including their ability for specific target recognition. It can be further enhanced by immobilizing nanomaterials as bio-elements such as (l-cysteine, DNA, *etc.*) at the electrode surface.^61^ Grasping the impact of these advancements on electrode performance is essential for enhancing the efficiency of electrochemical detection and monitoring. A thorough evaluation and comparison of nanomaterial-based sensors across various electrochemical techniques is vital to fully appreciate their advantages.

## 5. Figures of merit of the nanomaterial based electrochemical sensors

Electrochemical sensors utilizing nanomaterials have attracted significant interest and are frequently evaluated based on their figure of merits, which stem from their distinctive properties such as extensive surface area, numerous binding sites, enhanced catalytic activity, high porosity, and notable surface energy. The diminutive scale of nanostructured materials contributes to the thermodynamics of heterogeneous reactions and the kinetics of transport at the electrode interface, thereby facilitating mass and charge transfer. Additionally, these materials enable dimensional alterations through phase transitions and chemical reactions.^62^ These advanced electrodes provide a viable platform for detecting environmental pollutants due to their cost-effectiveness, sensitivity, rapid response times, ease of miniaturization, and capacity for functionalization with selective binding agents.^63^ It is essential to comprehend how these innovations influence electrode performance to optimize the efficacy of electrochemical detection and monitoring. The figures of merit for electrochemical sensors used in the detection of anticancer agents and antibiotics across various media are presented in Table 2 and 3.

**Table 2:** Performance of voltammetric techniques for the sensing of anticancer drugs

S. No	Analyte	Method	LOD (nM)	Linear range	Oxidation/Reduction peak potential (V)	Modifier	Electrode	Matrices	Ref.
1	Tamoxifen	CV	0.175	3–30 nM		PANI/HRP	Pt	Tablets	64
2		DPASV	0.08	1.3–16 μM		Bismuth film	GCE	Serum	65
3		DPV	0.132	0.2–40 nM	1.0	CeO_2_–MWCNTs	CCE	Human serum samples	66
4		SWV	1.81	10–60 nM	0.420	NiO	CPE	Human serum samples	67
5		DPV	25	0.1–200 μM	1.078	AI_2_O_3_@C/V_2_O_5_	CPE	Tablets	68
6	Etoposide	AdSDPV	5.4	0.02–2.0 μM	0.42	MWCNT	GCE	Pharmaceutical formulation	69
7		DPV	5	0.2–10 μM		CQDs	GCE	Real samples	70
8		DPV	2.8	0.5–10 μM		MIP	GCE	Biological fluids	71
9		CV	0.0016	0.5–5 nM	0.420	PLY-MWCNTs	GCE	Human blood samples	72
10		CV	4	0.01–1 μM	0.34	Oxime(i)	GCPE		73
11		DPV	2.7	0.04–120 μM	0.380	CEZOLLNPs	CPE	Urine and human serum	74
12	5-Fluorouracil	DPASV	0.825	0.1–0.17 μM	1.288	MIP	Ag	Pharmaceutics and blood plasma	75
13		SWV	0.4	1.0 nM –520 μM	0.125	Pt-SWCNTs/nH3MHP	CPE	Aqueous solution	76
14		SWV	0.5	0.001 to 10.0 μM		GQD/BPBr	CPE	Pharmaceutical samples	77
15		DPV	12	1–13 μM	1.17	Poly(GLY)	PGE	Real samples	78
16		DPV	10	0.1–40 μM	1.125	CeO_2_–CuO	CPE	Human urine samples	79
17	Doxorubicin	CV	3.67	0.09–7.36 μM	0.631	MWCNTs	Pt	Human plasma	80
18		CV	0.01	0.03–10 nM	0.55	Poly(Azure B-proflavine)	GCE	Blood serum	81
19		CV	0.1	0.1 μM–0.1 nM		Poly(Azure B)	GCE	Human serum	82
20		LSV	0.00063	0.01 nM–1 μM	−0.8	AuNPs/*f*MWCNTs	GCE	Pharmaceutical formulation	83
21		AdSDPV	2.5	0.01–9.5 μM		AuNRDs/1T-MoS_2_	SPE	Human serum	84
22		DPV	0.153	0.0003–0.014 μM	+0.390 and +0.750	GRQD	CNTPE	Water	85
23		DPV	14	30–750 μM		mSiO_2_/MWCNTs	GCE	Real samples	86
24	Imatinib	DPV	6.3	30 nM–0.25 μM	+1.2		BDDE	Human urine samples	87
25		DPV	0.4	0.0017–0.8500 μM	0.84	Fe_3_O_4_@MWCNTs@PANNFs)	CPE	Urine samples	88
26		DPV	0.6	0.002–100 μM	0.78	TbFeO_3_/g-C_3_N_4_	GCE	Real samples	89
27		DPV	3	0.01–20 μM		Ta/Cd-MOFs/AuNPs	CPE	Plasma	90
28		DPV	1.2	0.02–11.85 and 11.85 94.20 μM	0.812	CuFe_2_O_4_/ZIF-8@GQDs	GCE	Biological, pharmaceutical samples	91
29		DPV	7.3	1–300 μM	0.37	Chitosan/rGO	GCE	Human serum, urine samples	92
30	Mebendazole	ASDPV	10.5	0.02–1 μM	0.8	GNS–CNS/CS	GCE	Aqueous media	93
31		SWAdSV	19	0.06–3 μM	0.95	CNTs	GCE	Biological samples	94
32		DPV	4	0.01–1.5 μM	0.85	Fe–NCNF/MIP	GCE	Tape water, river water	95
33		DPV	25.81	0.3 μM–90 nM	1.27	GaN-PANI-PPy	GCE	Liquid samples	96
34	Carboplatin	DPV	4310	13.5–1350 μM	0.6		SPCE	Human urine	30
35	Cisplatin	DPV	4600	14.5 μM–0.1 mM	−0.1	MWCNT-COOH/SDS	SPCE	Human urine	97
36		DVASV	90	0.2–110 μM		GQDs-thio	npGCE	Biological fluids	98
37		DPV	4400	6.0–180 μM	−0.3	BiNPs/Gr	GCE	Human serum	99
38	Oxaliplatin	DPV	0.04	0.014–503 nM	−0.1	MIP-Ag@Cu-BDC/N-CNTs	GCE	Human serum	100
39		DPV	60	0.1–170 nM	0.3	Aptamer/AuPd NPs@ rGO/MWCNTs	GCE	Urine samples	31
40		DPV	10	10–2030 μM	0.5	g-C_3_N_4_/TiO_2_	GCE	Urine samples	101

## 6. Application of voltammetric techniques in drug analysis

### 6.1 Anticancer drugs

Anticancer therapies are generally prescribed to stop the cancer cells from multiplying and spreading. They may potentially damage normal cells and tissues causing side effects like tiredness, nausea, hair loss, and in some circumstances harm to key organs like the heart or liver. Numerous studies have been conducted on the electrochemical analysis of anticancer drugs utilizing various modified electrodes, with several examples outlined below:

#### 6.1.1 Tamoxifen (TMX)

**Fig. 2:**
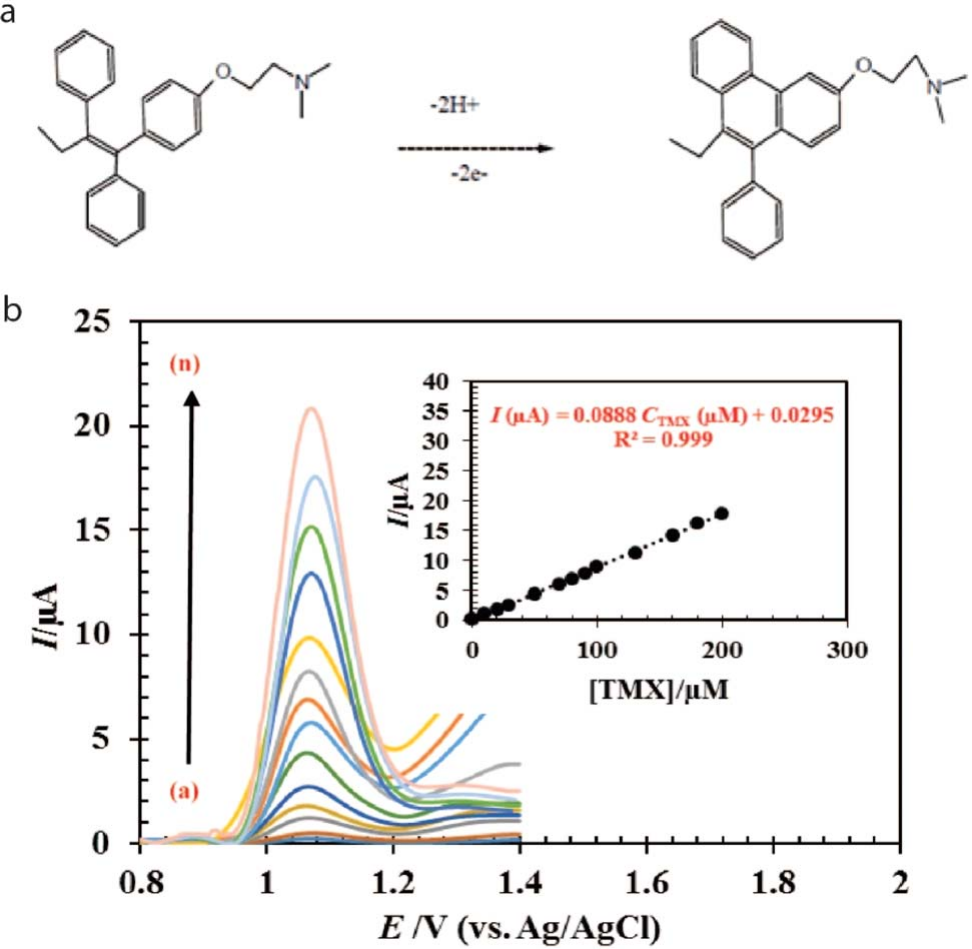
(a) Oxidation mechanism of TMX and (b) its corresponding DPV signal recorded at AI_2_O_3_@C/V_2_O_5_/CPE (inset, represents the linear range of 0.1 to 200 μM under optimized conditions.^68^ Reproduced with permission from ref. 68. Copyright (2022) *Microchem. J.*

Since its discovery in 1967, tamoxifen (TMX) has been employed in the prevention and treatment of breast tumor in women as well as breast cancer in general. Mood fluctuation, dry skin, nausea, bone pain, constipation, and eyesight issues are the most frequent adverse effects of using the drug. Consequently, determining tamoxifen in blood may be beneficial for curing these side effects for the patients. Thus, HRP-polyaniline modified Pt electrode was used for the cyclic voltammetric study of tamoxifen and the detection limit (LOD) was noted to be 0.073 ng mL^−1^. Furthermore, the stability of the sensor was reported by using the modified electrode at different time intervals for 10 days. The modified electrode was found quite stable with an RSD value of 1.01%.^64^ In a separate study to determine the trace quantity of TMX, a new differential pulse anodic stripping voltammetry method (DPASV) was developed to investigate the relationship between the tamoxifen concentration and the decline in peak amplitude of Bi oxidation on glassy carbon electrode (GCE) and exhibited a detection limit of 3.1 × 10^−5^ μg mL^−1^.^65^ Another study reported a modified electrode utilizing vanadium oxide deposited on glassy carbon electrode, which demonstrated a significant adsorption capacity for TMX, facilitating an oxidation reaction through electron transfer kinetics that involved two electrons and two protons. This process yielded an electrochemically inactive cyclized byproduct (diphenylphenanthrene) with LOD of 389 nM as determined by differential pulse voltammetry (DPV).^102^ Moreover, Shafaei *et al.* successfully synthesized a carbon ceramic electrode (CCE) modified with MWCNTs and cerium oxide (CeO_2_–MWCNTs/CCE) and found that the current electrode had potential towards detection of TMX drug up to 0.132 nM in serum samples.^66^ In a separate study researchers exploited nickel oxide nanoparticles as a modifying agent for carbon paste electrode (CPE) by using square wave voltammetry for the determination of TMX in human serum, achieving an impressive detection limit of 1.81 ± 0.35 nM.^67^ In a recent breakthrough, a modified carbon paste electrode (Ni(OH)_2_–NaY/CPE) doped with nickel nano zeolite sodium achieved a 0.65 μM detection limit of TMX *via* DPV. The existing sensor featured a substantial specific surface area and an intercrystalline mesoporous architecture, which facilitated the electrocatalytic oxidation of TMX by granting access to the active Ni^2+^ centers.^103^ Another study on the same technique was reported using CPE with vanadium oxide embedded within alumina forming a core–shell structure (AI_2_O_3_@C/V_2_O_5_/CPE) which exhibited higher sensitivity towards monitoring of TMX with an LOD of 0.025 μM than the previous reported ones due to synergistic effect of V_2_O_5_ and Al_2_O_3_@C that enhanced electron transfer rate through oxidation mechanism as represented in Fig. 2.^68^ The attachment of nanoparticles to the surface of carbon paste electrodes enhances electron transfer from TMX, attributed to their catalytic characteristics, while simultaneously releasing hydrogen atoms. This process results in the generation of an oxidized product, which is indicated by an irreversible peak in differential pulse voltammetry analysis.

#### 6.1.2 Etoposide (ETO)

The Food and Drug Administration of the United States (US) has licensed etoposide (ETO) commonly referred to as V-16, as an anticancer medication for the treatment of various types of tumors. It causes breakage of DNA strands and damages malignant cell DNA. It is highly desirable to detect the etoposide drug level in biological samples. Numerous researchers have implemented different voltammetric techniques for its detection. A GCE modified with MWCNTs has been reported for the sensing of ETO *via* AdSDPV, which showed a detection limit of 5.4 nM under optimized conditions (using Britton Robinson-BR buffer and pH 6.0). The altered electrode exhibited a current response that was approximately 25 times greater than that of the unmodified GCE. This enhancement is attributed to the significant adsorption capacity and extensive surface area of MWCNTs.^69^ In a new study by Aliakbarinodehi *et al.* screen printed electrode (SPE) modified with MWCNTs (MWCNTs-SPE) was utilized for the cyclic voltammetric monitoring of ETO drug with an LOD of up to 1.52 ± 0.89 μM, while a far more superior performance was found for gold nanoparticle decorated SPE (AuNPs-SPEs) with an LOD value of 1.29 ± 0.48 μM.^104^ In another study by Nguyen *et al.* 5 nM detection limit of ETO in real samples *via* DPV was reported using quantum dot modified GCE (CQDs/GCE). This study demonstrated that carbon quantum dots serve as an effective modifier, attributed to the presence of oxygen-rich groups in their structure, which enhanced the catalytic response.^70^ In addition to previous studies, the detection of ETO by employing the same technique was reported based on molecularly imprinted polymer modified GCE (MIPGCE) which showed a detection limit of 2.8 × 10^−9^ M with high reliability and sensitivity of the current method.^71^ Furthermore, a group of researchers successfully fabricated a film of poly (l-lysine) and MWCNTs integrated GCE (PLY-MWCNTs/GCE) which displayed excellent response towards the drug ETO with a very low detection limit of 1.6 × 10^−11^ M in human blood.^72^ Moreover, scientists investigated the cyclic voltammetric sensing of ETO drug using oxime(i) modified glassy CPE, which possessed a nanomolar level detection limit of 4.0 nM.^73^ Another detection at the nanomolar level, with an LOD of 2.7 nM was successfully achieved using DPV based on zinc oxide-lapis lazuli nanocomposite modified CPE. The remarkable electrocatalytic properties of the functionalized sensor can be ascribed to its elevated charge transfer rate and a greater number of active binding sites.^74^

**Fig. 3:**
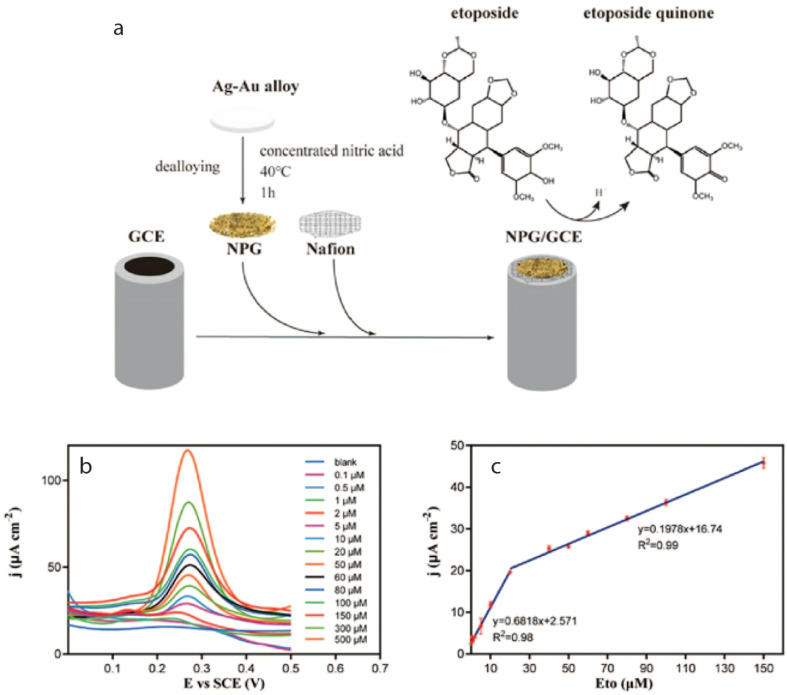
(a) Shows the fabrication of the sensor (NPG/GCE) and (b) corresponding DPV profile of ETO at an oxidation peak potential of 0.3 V (c) with two linear ranges of 0.1–20 μM and 20–150 μM.^106^

Most recently, a group of researchers utilized pencil graphite electrode (PGE) to monitor ETO drug up to 0.156 μM under optimized conditions such as BR buffer at pH 3.0, containing anionic surfactant of 0.5 mM *via* SWV.^105^ Subsequently, a modified GCE modified nanoporous gold (NPG/GCE) using DPV was explored, which showed an ETO detection limit of 20 nM in biological samples. It was anticipated that the oxidation of phenolic hydroxyl groups on ETO would lead to an increase in the production of ETO quinone, facilitated by NPG, as illustrated in Fig. 3, within the electrochemical sensors designed for the detection of phenolic compounds.^106^

#### 6.1.3 5-Fluorouracile (5-FU)

It is an uracil derivative anticancer drug that forms different adverse metabolites when present inside the body, so it is necessary to control and monitor its dosage. Herein, Hua *et al.* used MWCNTs/bromothymol blue (BTB) modified GCE for the detection of 5-fluorouracil (5-FU). The LOD for this drug was found to be 0.267 μM by CV. The modified electrode was also tested for its stability and it offered an RSD value of 5.4% over a time range of 15 days.^107^ Moreover, a group of researchers succeeded in the fabrication of a MIP-tuned Ag electrode which exhibited great potential towards the determination of a 5-FU drug with an LOD of 0.33 ng mL^−1^ in pharmaceuticals and blood plasma using DPASV.^75^ In another study, the quantification of the drug 5-FU was conducted using SWV and LSV. The SWV method demonstrated a superior limit of detection of 0.07 μM compared to LSV, attributed to the low interfacial resistance and enhanced charge transfer rate of the developed sensor utilizing zinc ferrite magnetic nanoparticles and ionic liquids modified carbon paste electrode designated as ZnFe_2_O_4_/MNPs/IL/CPE.^108^ Another group of researchers reported DPV detection of 5-FU up to 0.66 μM using a simple electrochemical probe based on CPE tuned with porphyrin-capped Au nanoparticles. The rapid electron transfer rate and efficient electrocatalytic performance of the modified electrode can be attributed to the superior conductivity and extensive surface area of gold nanoparticles present in the sensor.^109^ In addition, the fabrication of Ag nanoparticles incorporated polyaniline nanocomposite modified PGE (AgNPs@PANI/PGE) was successfully achieved, which exhibited super sensitivity towards the sensing of drug 5-FU with an LOD 0.06 μM using the same electrochemical technique in serum samples.^110^

Niazazari and coworkers examined the square wave voltammetric determination of the drug 5-FU by successfully fabricating a modified CPE using platinum incorporated SWCNT (Pt-SWCNTs) added with a *n*-hexyl-3-methylimidazolium hexafluorophosphate (*n*H3MHP) binder and found that the modified sensor performed better than other modified CPEs (CuO/IL/CPE and GQD/BPBr/CPE) in terms of detection limit of up to 0.4 nM.^76,77,111^ Most recently, a group of researchers fabricated a novel sensor based on 2D hexagonal boron nitride nanocomposite encapsulated in polythiophene-modified GCE (PTh/h-BN/GCE) for the monitoring of 5-FU using DPV and noticed that the modified sensor exhibited great sensitivity up to 0.02 μM.^112^ In a separate study, PGE modified by electro-polymerization of glycine (poly(GLY)/PGE) was developed which possessed an outstanding response towards the determination of 5-FU showing a detection limit of 0.012 μM using the same method.^78^ Furthermore, Vazan and his coworkers reported a LOD of 0.01 μM using cerium oxide-copper oxide nanocomposite (CeO_2_–CuO) modified CPE by DPV. The sensitivity and selectivity of modified sensor was attributed to oxygen exchange capability of CeO_2_ and its synergistic effect with CuO which facilitated the redox reaction and catalytic behavior. Additionally, 5-FU forms a complex with Cu^2+^ ions, which adsorbs at the surface of the electrode for improving the current response of the drug.^79^

#### 6.1.4 Doxorubicin (DOX)

**Fig. 4:**
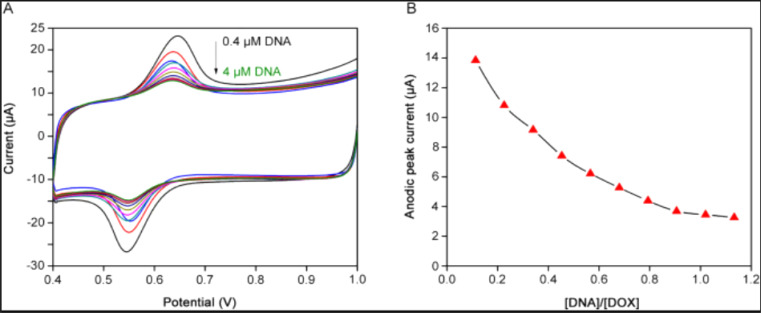
(A) CV profile of DOX at MWCNTs/Pt electrode following the successive insertion of DNA. (B) and the corresponding reduction of peak current confirming the Drug – DNA adduct.^80^

**Fig. 5:**
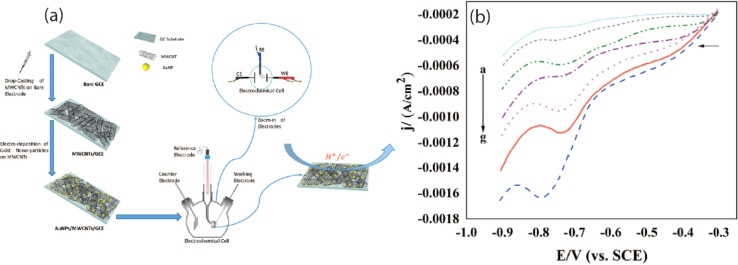
(a) Fabrication of MWCNTs-COOH/AuNPs/GCP and (b) corresponding LSV response of reduction of 5 μM DOX in 0.1 M PBS of pH 7.^82^

It is one of the anticancer drugs, frequently employed to treat breast cancer. However, despite its benefits, DOX causes several health issues. For the minute level sensing of DOX, a novel and efficient renewable film electrode based on silver amalgam (Hg (Ag)FE) was successfully fabricated and found that the current sensor showed an LOD of 0.08 μg mL^−1^ and 1.50 ng mL^−1^ using SWV and AdSWV respectively.^113^ Similarly, Hajian and his coworkers succeeded in the synthesis of MWCNTs modified Pt electrode, which was utilized for the cyclic voltammetric monitoring of DOX with a LOD of 0.002 μg mL^−1^. The presence of DNA was found to reduce the peak current of DOX at the constructed sensor, attributed to the drug's binding with DNA and the intercalation mechanism, which leads to a decreased diffusion coefficient, as illustrated in Fig. 4.^80^ In addition, researchers reported poly(Azure B-Proflavine) and poly(Azure B) modified GCE which showed detection limits of 0.01 nM and 0.1 nM respectively using the same method. Minor fluctuations in the current were observed during the motoring of DOX with a modified electrode, likely attributable to the hydrophobic characteristics of the analyte or steric hindrance that impacts its adsorption on the copolymer layer. This reduced activity may be advantageous when the sensor is utilized for drug analysis.^81^ Another study explored linear sweep voltammetry for the determination of DOX using graphite-based SPE showing LOD of 1.6 μg mL^−1^ and Au nanoparticle incorporated carboxylic acid functionalized MWCNTs functionalized GCPE with an LOD of 6.3 × 10^−12^ M respectively. The reduction peak observed at −0.8 V for 5 μM DOX is associated with the reduction of the quinone moiety, which entails a two-electron transfer at the constructed fMWCNTs/AuNPs/GCP. The linear sweep voltammogram illustrating this process is presented in Fig. 5.^82^ Most recently, investigators studied an electrochemical sensor made with molybdenum disulfide incorporated Au nanorods modified SPE (AuNRDs/1T-MoS_2_/SPE) using AdSDPV which showed great sensitivity towards the sensing of DOX up to 2.5 nM.^84^

In one particular study, DOX was analyzed by using two electroanalytical techniques, CV and DPV with a detection limit of up to 1.12 μM and 0.39 μM, respectively at the surface of sodium dodecyl sulfate modified CPE (SDS/MCPE).^83^ Similarly, LOD of 1.53 × 10^−10^ M in water was detected at the surface of graphene quantum dots (GRQD)-CNT paste electrode *via* DPV.^85^ Another group of researchers reported the fabrication of mesoporous silica (mSiO_2_) embedded MWCNTs modified GCE and found that the current sensor exhibited nanomolar level detection of DOX up to 14 nM using the same electroanalytical technique. Several research groups are investigating the effective electro-oxidation of DOX by employing MWCNTs and mesoporous silica as modifiers. The MWCNTs serve as a conductive framework that enhances charge transfer during the oxidation process, while mesoporous silica functions as a host matrix to immobilize DOX molecules on its porous surface. The combined effects of these materials significantly enhance the overall performance of the sensor.^86^

#### 6.1.5 Imatinib (IMB)

Imatinib is an antitumor medication employed in the treatment of various tumors and leukemia. The detection of imatinib in biological fluids and pharmaceuticals is essential for monitoring therapeutic drug concentrations, conducting pharmacokinetic studies, and ensuring the quality control of medicinal products. In this regard, researchers have successfully investigated the electrochemical oxidation characteristics of imatinib for the first time using a boron-doped diamond electrode (BDDE) through DPV. They found that the current electrode showed a detection limit of 6.3 × 10^−9^ M in human urine samples. The reduction signals of IMB have previously been documented using hanging mercury electrodes; however, due to mercury's toxicity, it has been substituted with an environmentally friendly BDD material.^87^ Moreover, Rouhi *et al.* reported the cyclic voltammetric detection of IMB with a detection limit of 1.2 μM using Ni NPs modified CPE.^114^ In addition to previous studies, the determination of IMB *via* AdSSWV was reported using a unique sensor based on carboxyl functionalized MWCNTs modified SPCE which showed a detection limit of 7 nM with an RSD of ≤7% showcasing superior sensitivity and reliability of the detection method.^115^ In a separate study by Ghapanvari and his coworkers, they examined the DPV detection of IMB using Fe_3_O_4_ NPs incorporated MWCNTs/polyacrylonitrile nanofibers modified CPE that showed a nanomolar level detection limit of 0.4 nM. The electrocatalytic activity of Fe_2_O_3_ particles significantly induced the oxidation of IMB, leading to a shift in the peak and an increase in the current density of the current modifier.^88^ Similarly, it has been observed that various modified GCEs have been developed for the detection of IMB *via* DPV. Notably, a nanocomposite of terbium-doped iron oxide and graphitic carbon nitride on GCE demonstrated a remarkable detection limit of 0.6 nM, outperforming other modified electrode platforms such as (N, S-CDs/CNTD, CuMOFs/MWCNTs) using the same electrochemical technique. The synergistic influence of terbium iron oxide particles and graphitic carbon nitride improved overall performance by addressing the low conductivity challenge associated with graphitic carbon nitride.^89,116,117^

Mir *et al.* developed a Ta/Cd metal–organic framework integrated with gold nanoparticles on a modified carbon paste electrode. Their findings indicated that the current sensor exhibited significant electrocatalytic activity for the oxidation of the IMB drug, and DPV analysis demonstrated a high sensitivity for detecting IMB in plasma, achieving a limit of detection of 3 nM.^90^ Researchers also presented an electrochemical investigation of a metal–organic framework derived from a zeolitic imidazolate framework (ZIF), which included graphene quantum dots and copper ferrite nanoparticles modified glassy carbon electrode (CuFe_2_O_4_/ZIF-8@GQDs/GCE). This study utilized DPV and demonstrated a detection limit of 1.2 nM for IMB. The designed sensor was found to amplify the oxidation signal of IMB, attributed to the substantial surface area and conductive properties of graphene quantum dots, along with the synergistic interactions of the other components.^91^ A recent study has demonstrated a detection limit of 7.3 nM for the IMB drug utilizing a reduced graphene oxide/chitosan modified glassy carbon electrode (chitosan/rGO/GCE) through differential pulse voltammetry. The method also achieved a mean recovery rate ranging from 95.00% to 99.20%, with a relative standard deviation (RSD) between 3.10% and 4.49%, indicating the method's reliability.^92^

#### 6.1.6 Mebendazole (MBZ)

Methyl-(5-benzoyl-1*H*-benzimidazol-2-yl) carbamate, commonly known as mebendazole, is a synthetic and effective anthelmintic agent that targets cestodes and tapeworms. Research has demonstrated that mebendazole possesses significant anti-cancer properties, highlighting the need for precise and dependable detection methods for MBZ in various samples. In response to this requirement, a sensor was developed using carbon nanospheres and chitosan, enhanced with graphene nanosheets on a glassy carbon electrode. This modified sensor exhibited a limit of detection of 10.5 nM in aqueous solutions, attributed to the excellent film-forming properties and biocompatibility of chitosan.^93^ In another study, a detection limit of 0.6 μM for MBZ in pharmaceutical formulations was achieved through the use of LaFeO_3_ perovskite-type nanoparticles modified sonogel carbon paste electrodes (SNCPE) *via* DPV. The exceptional electrical conductivity and stability of LaFeO_3_ contribute significantly to its electrocatalytic properties and ensure its dependability for prolonged applications.^118^

In addition to previous studies, square wave adsorptive stripping voltammetry (SWAdSV) determination of mebendazole was reported using fCNT-modified GCE (fCNTs-GCE) which showed a detection limit of 19 nM. Its DFT study was also performed to investigate the type of interaction, electronic structure, and stable configuration of MBZ on fCNT.^94^ In another study, researchers explored the electrochemical detection of MBZ utilizing Fe-doped nitrogen-enriched carbon nanosheet frameworks (Fe–NCNF) integrated with MIP modified GCE. The study revealed that the sensor was capable of detecting MBZ with LOD of 4 nM. The incorporation of FeNPs solved the issue of poor electroconductivity of MIPs and enhanced the overall sensing ability of the sensor.^95^ Another group of researchers successfully fabricated an electrochemical sensor utilizing a nanocomposite of MWCNT and poly(*o*-anisidine) (POA) modified GCE. The differential pulse voltammetry analysis demonstrated that this sensor exhibited an enhanced catalytic current response, attributed to its superior charge transfer capability, with a detection limit of 0.4 μM for MBZ in actual samples.^48^ Similarly investigators have also reported the identification of the drug MBZ utilizing the same methodology, achieving a limit of detection of 2.581 × 10^−8^ M with a sensor constructed from a nanocomposite of gallium nitride-polyaniline-polypyrrole (GaN-PANI-PPy) modified glassy carbon electrode.^96^ The comprehensive performance evaluation of voltammetric techniques for the electrochemical assessment of various anticancer drugs is presented in Table 2.

### 6.2 Platinum based drugs in chemotherapy

Platinum-based drugs, including carboplatin, cisplatin, and oxaliplatin, are commonly employed in the chemotherapeutic treatment of cancer, as detailed in Table 2. Nonetheless, their clinical application is often compromised by adverse effects such as heightened toxicity, reduced sensitivity, and the development of drug resistance, necessitating careful monitoring. In this regard, electrochemical analysis of carboplatin has been conducted using screen-printed carbon electrodes (SPCE) in laboratory settings. The study revealed that the application of various conductive inks on plastic or ceramic substrates through screen printing offers advantages like cost-effectiveness, flexibility, and ease of surface functionalization. Differential pulse voltammetry analysis indicated the presence of carboplatin at 0.6 V, with a linear concentration range of 3.5 to 1350 μM and a detection limit of 1.6 μg mL^−1^ in artificial tears, saliva, and human serum. Additionally, another investigation focused on the electrochemical analysis of cisplatin using DPV on a modified screen-printed electrode incorporating fMWCNTs and sodium dodecyl sulfate, which was found to be crucial for improving drug response, achieving a detection limit of 4.6 μM in biological samples.^97^ Sodium dodecyl sulfate serves to enhance the drug's adsorption and stabilize MWCNTs. Gholivand *et al.* demonstrated a detection limit of 90 nM for cisplatin utilizing a modified nanoporous glassy carbon electrode integrated with graphene quantum dots and thionine. The DPASV analysis indicated that the current sensor exhibited high sensitivity in real samples, attributed to the interaction between cisplatin and thionine, which was further augmented by the distinctive properties of graphene quantum dots.^98^

Another group of researchers explored graphene modified GCE coated with bismuth nanoparticles to design selective and sensitive sensors for the detection of cisplatin. The bismuth nanoparticles serve to enhance surface area, electron transfer rate, and catalytic activity while graphene possesses characteristics such as large surface area, excellent chemical stability, and superior conductivity for supporting metal nanosomes. Electrochemical behavior was examined by DPV which showed oxidation of cisplatin in the linear range of 6 μM to 80 μM and a detection limit of 4.4 μM in human serum.^99^ Mahnashi and colleagues developed a MIP-modified GCE utilizing N-doped carbon nanotubes, silver nanoparticles, and copper metal–organic frameworks (MIP-Ag@Cu-BDC/N-CNTs/GCE). This electrode was employed for the DPV analysis of oxaliplatin, achieving a detection limit of 0.016 ng mL^−1^. It was found that the conductivity and anchoring points for both the polymer layer on the electrode surface were enhanced by combination of N-CNTs and Ag/Cu-BDC.^100^ Another group of researchers reported the detection of oxaliplatin up to 60 nM through an aptasensor that utilizes AuPd nanoparticles integrated with reduced graphene oxide and multi-walled carbon nanotubes, employing a similar methodology. Their findings revealed that the folding and conformational changes of the aptamer oligonucleotides hindered electron transfer, leading to a decrease in anodic peak current as the concentration of the analyte increased.^31^ In other study researchers identified a detection limit of 0.010 μM for oxaliplatin in urine and blood samples using DPV at the surface of a nanocomposite comprising g-C_3_N_4_ and TiO_2_ modified GCE. The combined effects of g-C_3_N_4_ and TiO_2_ significantly enhanced catalytic performance, as the layered structure of g-C_3_N_4_ contributed to an increased surface area, while TiO_2_ facilitated a higher electron transfer rate, thereby enabling precise monitoring of the drug.^101^

### 6.3 Antibiotic drugs

Antibiotics are pharmaceutical agents employed to treat bacterial infections. Potential side effects encompass allergic reactions, nausea, and abdominal discomfort, alongside the risks associated with inappropriate or prolonged usage, which can result in antibiotic resistance. This resistance diminishes their efficacy in addressing future health issues. Numerous substantial research initiatives have been undertaken to enhance the electrochemical monitoring of antibiotic medications, with several methodologies outlined in the following sections:

#### 6.3.1 Sulphadiazine (SUZ)

**Scheme 2:**
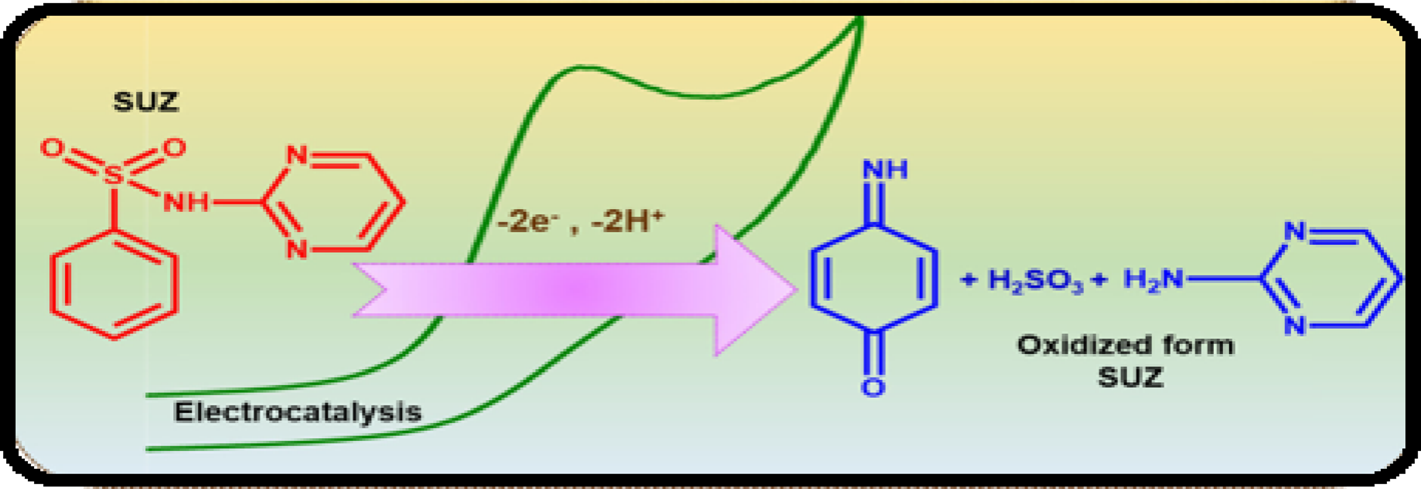
Electro-oxidation mechanism of sulphadiazine drug at Cu_2_Sb/SPCE.^122^

**Fig. 6:**
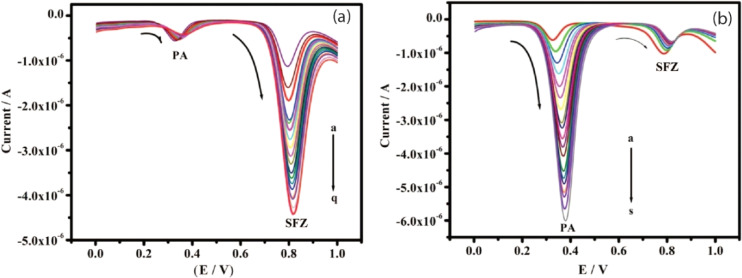
(a) DPV profile of SUZ in the presence of 0.1 mM paracetamol at CdO/CPE. (b) Voltammogram of paracetamol in the presence of constant 0.1 mM SUZ using the same modified electrode.^124^ Reproduced with permission from ref. 124. Copyright (2023) *Inorg. Chem. Commun.*

This medication serves as an antibacterial agent, effective in treating a range of infections, including toxoplasmosis, urinary tract infections (UTIs), and chancroid, among others. Nonetheless, concerns have emerged regarding the long-term use and excessive application of SUZ, which may lead to liver damage, genetic degradation, and chronic toxicity. In this context, a team of researchers has successfully developed a modified GCE that demonstrates significant capability in assessing SUZ, achieving LOD of 7.1 μM through cyclic voltammetry.^119^ In a separate study, adsorptive differential pulse cathodic stripping voltammetry (DP-CSV) monitoring of SUZ was performed by using a hanging mercury dropping electrode which exhibited LOD of 1.1 × 10^−9^ M in wastewater.^120^ Furthermore, Liu *et al.* investigated the DPV determination of SUZ in real samples up to 0.68 μM using MWCNT incorporated MIP modified GCE.^121^ In a separate study, a 70 nM detection limit of SUZ has been reported by using the same technique based on a novel copper antimony compound modified SPCE (Cu_2_Sb/SPCE). The findings indicated a significant surface area, reduced resistance coupled with enhanced conductivity, improved electrocatalytic performance, and greater electro-oxidation efficiency concerning the oxidation of the NH group in SUZ, which entails the transfer of two electrons and two protons. This enhanced electrocatalytic activity of SUZ can be attributed to the presence of Cu_2_Sb nanoparticles, as illustrated in the accompanying Scheme 2.^122^ SUZ followed an irreversible process with a lower oxidation potential at physiological pH of 7.2. Another group of researchers performed DPV analysis of SUZ and found that strontium tungstate (SrWO_4_) nanoflake modified GCE showed an LOD of 0.009 μM.^123^ Subsequently, researchers utilized electrochemical methods, specifically CV and DPV, for the determination of SUZ using a cadmium oxide-modified carbon paste electrode (CdO/CPE), which demonstrated a limit of detection of 0.216 μM. Furthermore, the modifier employed allows for the individual and selective monitoring of both SUZ and paracetamol, as illustrated in Fig. 6, due to its exceptional electrochemical properties.^124^ Most recently, Tb_2_(WO_4_)_3_ modified CPE was reported for the detection of SUZ *via* CV. The LOD was recorded to be 0.004 μM in real samples.^125^

#### 6.3.2 Metronidazole (MNZ)

**Fig. 7:**
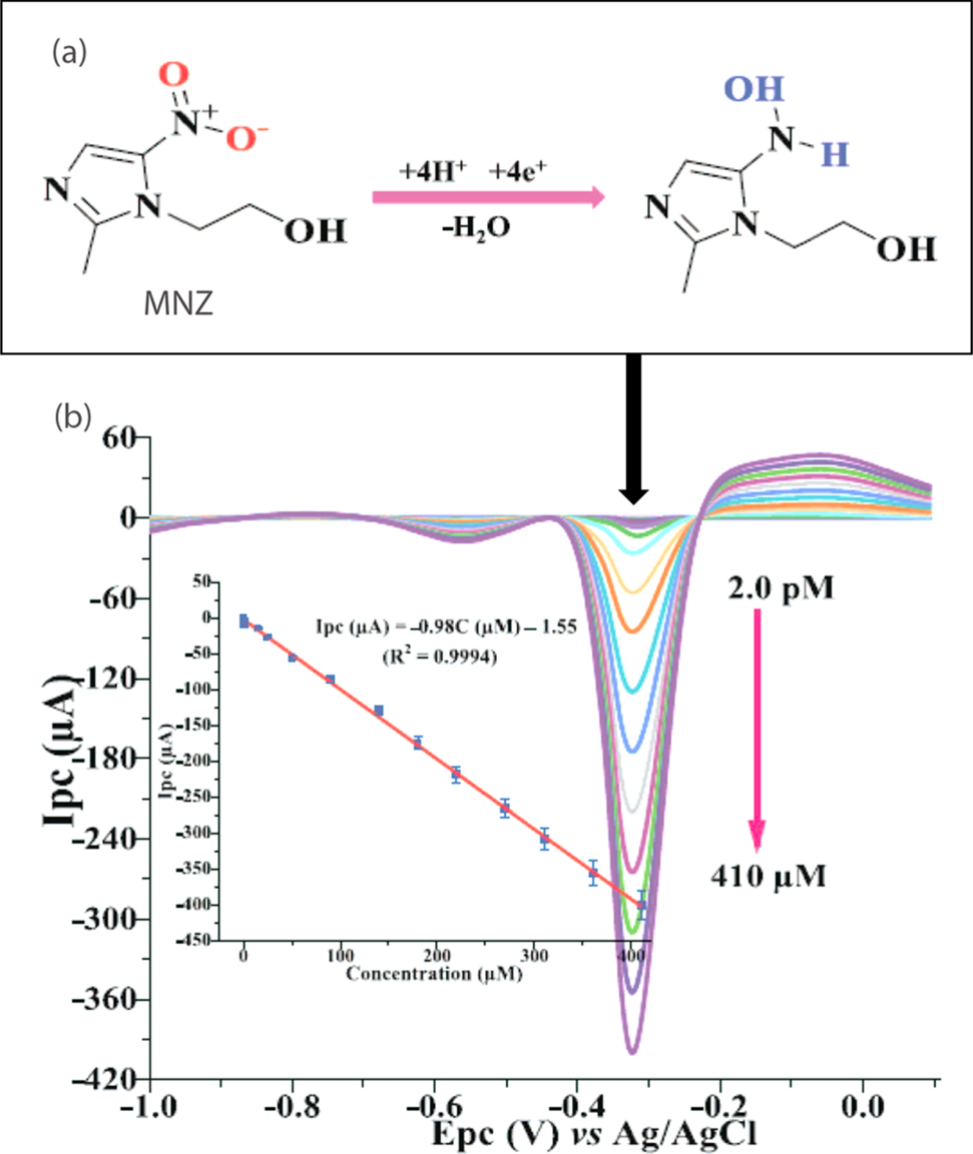
(a) Reduction mechanism and (b) the corresponding square wave voltammogram of MNZ at TA-Au–Ag-ANpM/r-GO/poly(glycine)/GCE within a potential range of −1.2 to 0.1 V, having reduction peak potential of – 0.3 V. The inset shows a linear relationship between the reduction peak current and concentration of MNZ.^133^ Reproduced with permission from ref. 133. Copyright (2024) *Chemosphere*.

This drug is used to treat infections caused by bacteria such as abscess, vaginosis, amebic liver, *etc.* However, it causes ataxia, peripheral neuropathy, seizures, and other toxic responses in the human body when the cumulative dosage of MNZ surpasses a particular limit. Numerous analytical procedures have been proposed for its monitoring. Chen *et al.* fabricated a unique sensor based on magnetic molecularly imprinted polymer (MMIP) modified GCE which showed improved sensitivity towards the evaluation of MNZ with a detection limit of 1.6 × 10^−8^ M by using DP-CSV.^126^ In another study, researchers utilized cystic acid incorporated graphene functionalized with poly(diallyl dimethylammonium chloride) modified GCE towards the determination of MNZ by using LSV which showed high sensitivity with a detection limit 2.3 nM.^127^ Similarly, a 0.1 nM detection limit has also been achieved with a three-dimensional Au nanotube modified carbon paste electrode through square wave anodic stripping voltammetry, demonstrating that the sensor's high sensitivity is attributed to the extensive surface area provided by the Au nanotube.^128^ In another study, an ultrasensitive detection of MNZ using a unique concept of support free electrochemical platform based on MIP modified with Au–Ag microrods was reported with a detection limit of 0.02 pM. The stability of the sensor was also tested for about 15 days and it offered reasonable stability with RSD value of 3.67%.^129^ In addition the determination of MNZ using LSV was conducted using porous carbon materials doped with triple atoms (N, S, and P) derived from biomass as an electrode sensor. This method demonstrated a limit of detection of 0.013 μM and achieved a mean recovery rate between 93.6% and 100.1%, with a relative standard deviation of 4.7%, indicating the method's reliability and applicability.^130^ Similarly, a 5 nM detection limit of MNZ has been reported by using heteroatom modified GCE with oxygen enriched graphitic carbon nitride (O-gCN) *via* DPV method in water samples. The challenge of low specific surface area in gCN was addressed through the incorporation of heteroatom dopants, which enhanced the electrocatalytic activity of the sensor.^131^ Another study on the same technique was reported using nanocomposite based on graphitic carbon nitride incorporated molybdenum disulfide (MoS_2_) modified GCE which showed a LOD of 0.099 μM for MNZ with an RSD value of 2.43%.^132^ Subsequently, ultra-sensitive sensor was successfully developed by integrating reduced graphene oxide and poly(glycine) with thermally annealed Au–Ag alloy nanoporous matrix at GCE surface (TA-Au–Ag-ANpM/r-GO/poly(glycine)/GCE). The square wave voltammograms presented in Fig. 7 identified MNZ in food samples, achieving a detection limit of 0.0312 pM. The electrode modifier's effectiveness is linked to the crystalline structure and distinctive porosity of the novel sensor material, which enhances sensitivity and increases surface area by removing grain boundaries.^133^

#### 6.3.3 Levofloxacin

**Fig. 8:**
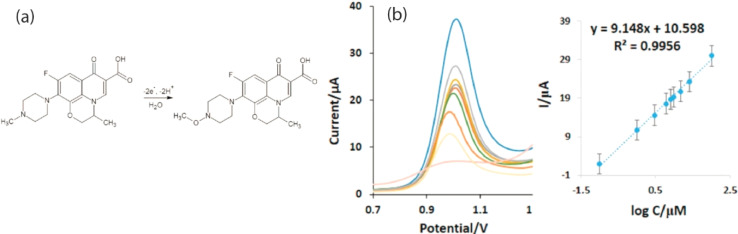
(a) Mechanism of levofloxacin and (b) the DPV voltammogram at varying concentrations from 0.1 to 100 μM using AuNPs/rGO/GCE.^138^ Reproduced with permission from ref. 138. Copyright (2019) *Microchem. J.*^138^

It is one of the quinolone-type medications, typically prescribed as an antibiotic with a wide spectrum for various types of human and animal illnesses. However, the widespread use of levofloxacin raises the chance of developing certain illnesses including pseudomembranous colitis, tendinopathy, heart disease, and muscle atrophy. In this context a modified GCE utilizing gold nanoparticles combined with a cerium oxide/gold nanofiber composite (AgNPs/CeO_2_–Au/GCE) was effectively developed, that detected levofloxacin at concentrations as low as 0.01 μM. The sensor exhibited a robust current response, a stable matrix, and an extensive surface area, as validated by DPV analysis.^134^ Similarly the detection limit of levofloxacin at 10 μM was established using the cyclic voltammetry method with a boron-doped diamond electrode.^135^ Lei Han and his colleagues investigated the linear sweep characteristics of levofloxacin utilizing GCE with rGO-based poly(*p*-aminobenzene sulfonic acid). The designed sensor outperformed other rGO-based electrodes, such as PDDA-rGO/AuNPs/GCE, in terms of sensitivity, demonstrating a broader linear range and LOD of 0.12 μM.^136,137^ Another group of researchers, studied the electrochemical response of poly(l-cysteine) incorporated Au nanoparticles and rGO modified GCE using DPV and found that the current sensor exhibited high sensitivity towards levofloxacin with LOD of 3.0 × 10^−12^ M under optimal settings such as 0.1 M PBS and a pH of 6.5. The l-cysteine is known to create numerous active sites for binding the analyte and enhance electron transfer. The determination of levofloxacin up to picomolar level at AuNPs/rGO/GCE *via* DPV is shown in Fig. 8.^138^ Levofloxacin undergoes irreversible oxidation at the electrode, characterized by a distinct peak resulting from a stable matrix created by rGO on l-cysteine, which is utilized for the incorporation of AuNPs. The combination of these elements significantly improves both the sensitivity and selectivity for the drug. The proposed oxidation mechanism involves the transfer of two electrons and two protons, while the anodic peak observed within the potential range of 0.7 to 1.2 V is linked to the irreversible oxidation of the electron-donating piperazine group, leading to the formation of the drug's oxidized form at the AuNPs/rGO/GCE, which is subsequently detected electrochemically.

#### 6.3.4 Chloramphenicol (CAP)

Chloramphenicol is a wide-spectrum antibiotic frequently used to cure infectious disorders in animals and humans because of its effectiveness against both Gram-positive and Gram-negative bacteria. A group of investigators reported the square wave analysis of CAP in human urine samples by using gold nanoparticles embellished magnetite particles stabilized with carboxymethyl cellulose (CMC) on a GCE (Au@Fe_3_O_4_-CMC/GCE) which showed high sensitivity towards CAP with an LOD of 6.6 × 10^−8^ M due to large surface area of Au shell nanoparticles in association with core iron nanoparticles which help overcome reactivity issues of Fe NPs.^139^ Later, another group of researchers fabricated an efficient sensor based on exfoliated porous carbon (EPC) modified GCE which showed a detection limit of 2.9 × 10^−9^ M, better than the previously reported one by using the same analytical technique. This was attributed to large surface area and enhanced dispensability of EPC.^140^ Another sensor based on nanohybrid material 3D CNTs/Cu nanoparticles with MIP modified GCE was reported to have LOD of 10 μM. The sensor was also found quite stable as it offered an RSD value of 0.99% when subjected to repeatability experiments for about 1 month using CV.^141^ Researchers utilized Ag nanoparticles and short chain –COOH functionalized MWCNTs modified GCE for the accurate and precise linear sweep stripping voltammetric evaluation of CAP with LOD of 0.049 μM in real matrices. The enhanced conductivity and increased surface area provided by gold nanoparticles in conjunction with fMWCNTs resulted in effective charge transfer between the GCE and the target compound.^142^ In a similar study, the detection of the same drug at concentrations as low as 9.84 nM was achieved using a GCE modified with MWCNTs and copper nanodendrites (CuNDs) through linear sweep voltammetry. This sensor demonstrated superior sensitivity compared to the previous one, attributed to the excellent conductivity and cost-effectiveness of copper nanoparticles relative to noble metal nanoparticles, which enhance the bonding with MWCNTs and overall catalytic efficiency.^143^ Another sensor identified CAP in milk samples with a limit of detection of 8.7 nM by employing differential pulse voltammetry at the GCE modified with nanoscale manganese oxide particles, attributed to their enhanced surface area, biocompatibility, and excellent chemical stability.^144^

#### 6.3.5 Ofloxacin

It belongs to the second generation of antibiotics having a significant effect against respiratory infections such as (pneumonia, bronchitis) along with infections of the skin and reproductive organs. For the low-level monitoring of ofloxacin, investigators presented a unique sensor based on nanocomposites (HPMαFP(1-phenyl-3-methyl-4-(2-furoyl)-5-pyrazolone) and polypyrrole (PPy)) modified GCE which showed a detection limit of 6.5 × 10^−8^ M *via* DPV in ophthalmic solution samples.^145^ Square wave adsorptive anodic stripping voltammetric (SWAdASV) analysis of ofloxacin at graphene oxide incorporated ionic liquid modified CPE (GO-IL/CPE) was reported which possessed a low LOD of 2.8 × 10^−10^ M in ophthalmic and human urine samples. It was observed that ionic liquid as a modifier enhanced heterogeneous electron transport and catalytic response in combination with nanomaterials like graphene.^146^ In a separate case study, decorated Pt–Au with rGO-modified GCE was designed to evaluate ofloxacin in human urine and tablets *via* DPV up to 0.05 μM.^147^ The alloying of Pt with Au was found to remarkably improve the electrocatalytic response of Pt for ofloxacin. Another sensor based on graphene-incorporated zinc oxide composite immobilized onto a GCE detected ofloxacin up to 0.33 μM in real samples using the same detection method.^148^ In another study, cyclic voltammetry was used for the detection of ofloxacin with LOD of 0.037 μM using GCE modified with a nanocomposite of Pt/KB/CD-MOFs. The Ketjen black (CB) in the modifier resolved the low conductivity issue of CD-MOFs and enhanced the overall efficiency in combination with Pt nanoparticles.^149^ Similarly, investigators studied the detection of ofloxacin using MXene-based composite modified GCE (nAu@Ti_3_C_2_T_*x*_/PABSA/GCE) which showed a detection limit of 3.7 × 10^−8^ M *via* DPV in real samples.^150^ Another document details the use of linear sweep voltammetry for the determination of ofloxacin in actual samples, achieving a limit of detection of 0.12 μM with an electrode modified by a nanocomposite of molybdenum disulfide and multi-walled carbon nanotubes.^151^

#### 6.3.6 Ciprofloxacin (CIP)

It is a 3rd generation antibiotic frequently prescribed for infections including urinary tract infections, chronic bacterium prostatitis, and respiratory tract. Voltammetric techniques are widely used for the detection of ciprofloxacin. Bagheri and colleagues indicated that the combination of MWCNTs and Fe_2_O_3_ nanoparticles led to the development of a magnetic MWCNT/MIP sensor, which exhibited improved electron transfer rates and overall catalytic performance. The DPV assessment of ciprofloxacin in biological fluids and pharmaceutical formulations utilizing this sensor revealed a limit of detection of 1.7 nM.^152^ Similarly, reports have shown nanomolar level detection of CIP using the same technique with an LOD of 5 nM at –COOH functionalized MWCNT modified BDD electrode in water samples. It was observed that functionalized CNTs greatly influence the electrochemical activity by facilitating enhanced interaction with the drug.^153^ In a separate study, cyclic voltammetric determination of CIP was reported using novel polyaniline film decorated β-cyclodextrin with MWCNT modified GCE. The analysis showed that the tuned electrode possessed a large surface area that detected CIP with LOD of 50 nM in water samples.^154^ The assessment of CIP and the influence of surface termination on electrochemical performance was conducted using Boron doped diamond powder (BDDP) as a reference. Oxygen-terminated and hydrogen-terminated BDDP (O-BDDP and H-BDDP) were combined with resin to create an ink suitable for application on printed electrodes. The results from linear sweep voltammetry indicated that the oxygen-terminated BDDP printed electrode exhibited a more pronounced anodic peak and a greater current density, achieving a limit of detection of 0.59 μM and a mean recovery rate of 107% in urine samples.^155^ In another study, researchers examined the square wave signature of CIP by utilizing a PGE and found that the current electrode possessed an LOD of 5.6 μM in pharmaceutical formulations due to unique characteristics of PGE such as high stability, broad potential range, and strong analyte adsorption.^156^ Another group of researchers modified GCE with 2-(hydroxymethyl)thiophene (p(2 TM)) and discovered that p(2 TM) on the GCE exhibits significant electrocatalytic activity for ciprofloxacin. Additionally, square wave stripping voltammetric analysis demonstrated its high sensitivity to CIP, achieving a limit of detection of 7 nM in urine samples.^157^ Further, analytical capabilities of various voltammetric techniques for the determination of antibiotics are presented in Table 3.

**Table 3:** Electrochemical methods and platforms for the evaluation of LODs of various antibiotics

S. No	Analyte	Method	LOD (nM)	Linear range	Potential (V)	Modifier	Electrode	Matrices	Ref.
1	Sulphadiazine	DP-ACSV	1.1	3.7 nM–0.1 μM			HMDE	Wastewater	120
2		DPV	70	0.09–818.1 μM	0.96	Cu_2_Sb	SPCE	Cream samples	122
3		DPV	9	0.05–235 μM	0.93	SrWO_4_	GCE	Real samples	123
4		CV	4	0.01–10 μM	0.85	Tb_2_(WO_4_)_3_	CPE	Real samples	124
5	Metronidazole	LSV	2.3	10 nM–1 μM	−0.7	Poly(diallyl dimethyl ammonium chloride)	GCE	Urine, lake water	127
6		SWV	0.1	1 nM–2 μM	0.2	3D GNT	CPE		128
7		CV	0.000027	80 nM–1 μM	0.3	MIP/NPAMRs	MIP/NPAMRs	Fish tissue, drug tablets	129
8		LSV	13	0.1–45 μM		NSP-PC	GCE	Pharmaceuticals, milk	130
9		DPV	5	0.01–2060 μM	−0.65	O-gCN	GCE	Water samples	131
10		SWV	0.0000312	2.0 pM–410 μM	−0.33	TA-Au–Ag-ANpM/r-GO/poly(glycine)	GCE	Food samples	133
11	Levofloxacin	DPV	10	0.03–10 μM	0.9	AgNPs/CeO_2_–Au	GCE	Human urine, blood serum	134
12		LSV	120	0–50 μM	1.0	Poly(*p*-ABSA)-rGO	GCE	Human urine samples	136
13		LSV	3900	10 μM–0.2 mM		Au/PDDA/rGO	GCE	Pharmaceutical tablets	137
14		DPV	0.00030	0.01 nM–0.1 mM	0.9	Poly(l-cys)/AuNPs/rGO	GCE	Blood serum	138
15	Chloramphenicol	SWV	660	2.5–25 μM	−0.7	Au@Fe_3_O_4_-CMC	GCE	Human urine samples	139
16		SWV	2.9	10 nM–1 μM	−0.1	EPC	GCE	Honey	140
17		LSSV	49	0.3–229 μM		Short-MWNTs-COOH/AgNPs	GCE	Real samples	142
18		LSV	9.84	0.15–12 μM	−0.63	CuNDs/MWCNTs	GCE	Water samples	143
19		DPV	008.7	0.005–480 μM	−0.63	Mn_3_O_4_	GCE	Milk	144
20	Ofloxacin	SWAdASV	0.28	7 nM–0.7 μM	0.99	GO-IL	CPE	Ophthalmic, human urine samples	146
21		DPV	50	0.08–10 μM	0.93	Pt–Au/rGO	GCE	Human urine, tablets	147
22		DPV	330	1–100 μM		ZnO/GR	GCE	Real samples	148
23		CV	37	0.08–100 μM		Pt KB/CD-MOFs	GCE	Serum	149
24		LSV	120	0.29–0.82 μM	0.85	MoS_2_/MIP		Real samples	151
25	Ciprofloxacin	DPV	1.7	0.005–0.85 μM		MWCNT/MIP	CPE	Biological fluids, pharmaceutical formulation	152
26		DPV	5	0.005–0.05 μM	1.5	COOH-MWCNT	BDD	Water samples	153
27		CV	50	10–80 μM	0.95	PANI-β-CD/*f*MWCNT	GCE	Water sample	154
28		SWSV	7	0.1–200 μM	1.1	*p*(2 TM)	GCE	Urine samples	157
29	Cefixime	SW	570	2.2–1.3 μM	1.25		BDDE	Pharmaceutical formulations	158
30		DPV	35	0.06–10 μM	0.85	IL/CoFe_2_O_4_/rGO(1-ethyl-3-methylimidazolium)	CPE	Biological samples	159
31		DPV	0.5	2.0–1000 μM	0.8	Cu(Him)_2_/IL	CPE	Biological samples	160
32		SW-CSV	26	0.1–1.60 μM		AgNP	APME		161
33	Amoxicillin	SWV	193	0.5–80 μM	0.5		RGOnS	Water samples	162
34		SWV	9.9	10–150 μM	0.9	Poly(AHNSA)	GCE	Tablet formulation	163
35		DPV	0.29	5 nM–0.9 μM	0.1	MIP/GO	GCE		164
36		DPV	158	0.2–20 μM	0.6	Zn/NPh	CPE	Water samples	165

### 6.4 β-Lactams

Apart from the antibiotics discussed in the previous sections, beta-lactams, specifically cefixime and amoxicillin, are utilized for the treatment of bacterial infections listed in Table 3. The distinct chemical and pharmacological properties of various beta-lactams arise mainly from the side chains attached to the beta-lactam ring. Nevertheless, the overuse of beta-lactams can lead to adverse effects such as bleeding, nausea, rashes, and vomiting, necessitating careful monitoring. For example, a detection limit of 5.9 × 10^−7^ M for the drug cefixime was observed at BDDE using SWV. The BDDE facilitated highly sensitive and selective detection of cefixime. By analyzing the oxidation peak current at 1.25 V in relation to cefixime concentration, the electrochemical behavior was evaluated, indicating that the process is irreversible.^158^ In a study conducted by Darabi *et al.* a modified CPE was utilized as a sensor for cefixime, incorporating a synthesized rGO/CoFe_2_O_4_/IL (1-ethyl-3-methylimidazolium) nanocomposite. The selection of CoFe_2_O_4_ was based on its excellent electrical characteristics when paired with rGO, along with the rapid electron transfer capabilities of the ionic liquid, 1-ethyl-3-methylimidazolium chloride, recognized for its high conductivity. The differential pulse voltammetry analysis revealed a detection limit of 0.035 μM at an oxidation peak potential of 0.85 V.^159^ Another sensor made of CPE modified with Cu(Him)_2_ and IL was also used to monitor cefixime *via* DPV. The electrode modifier led to significant enhancement in anodic peak current of cefixime with a detection limit of 0.5 nM.^160^ Another group of researchers synthesized Ag nanoparticles amalgam paste microelectrode (AgNP-APME) and investigated the electrochemical reduction of cefixime by CV, SW, and DP cathodic stripping voltammetry. Within the potential range of −1.2 to −0.4 V, CV revealed a single reduction peak, whereas DP-CSV and SW-CSV displayed two distinct peaks, which were attributed to the reduction of unsaturated C=N (ozo-methine) and C=C groups, corresponding to four and two electron transfers, respectively. A detection limit of 2.6 × 10^−8^ M was obtained by SW-CSV in the linear concentration range of 0.1–1.60 μM.^161^

Amoxicillin (AMX) was identified at a detection limit of 0.193 μM using a reduced graphene oxide nanosheet-based electrode (rGOnS) through square wave voltammetry. The sensor exhibited an oxidation signal for AMX that was tenfold higher compared to an unmodified graphite electrode, attributed to extensive surface area, rapid electron transfer rate, and enhanced stability of the modified electrode.^162^ Another group of researchers reported SWV analysis of AMX in the linear range of 10–150 μM at modified GCE based on poly(4-amino-3-hydroxynaphthalene-1-sulfonic acid) and found that peak current response linearly increased with the addition of AMX concentration with a detection limit of 9.9 nM.^163^ MIP-incorporated graphene oxide-modified GCE was synthesized to analyze AMX *via* DPV, the incorporation of graphene oxide effectively addressed the issues of low conductivity and reduced sensitivity in MIP, attributed to its extensive surface area and enhanced conductivity. This modification achieved a limit of detection of 0.29 nM at an oxidation peak potential of 0.1 V.^164^ Another study reports electrochemical analysis of amoxicillin at zinc integrated hydroxyapatite modified CPE. The designed sensor analyzed amoxicillin by oxidative mechanism and the sensor material was pre-activated by using UV radiations that enhanced its electrocatalytic response. The oxidation mechanism involved two protons and two electrons at Zn/NPh/CPE with a detection limit of 1.58 × 10^−7^ M.^165^

## 7. Critical analysis of the electrochemical techniques

Various voltammetric techniques such as CV, LSV, DPV and SWV each offer unique advantages and limitations. CV is often favored for its versatility, efficiency, and ability to provide mechanistic insights, although it faces challenges such as matrix interference and sensitivity issues. LSV is appreciated for its simplicity and low detection limit, yet it has a limited dynamic range and lower sensitivity compared to DPV and SWV. DPV is recognized for its straightforward application, low background current, and usefulness in pharmacodynamics and pharmacokinetics, although it can be time-intensive. Conversely, SWV is noted for its high sensitivity, yielding peak currents up to four times greater than those of DPV, and it is quicker and suitable for electrode kinetic studies. However, it requires complex data analysis and careful parameter optimization. A summary of the primary benefits and drawbacks of these voltammetric techniques is provided in Table 4.

**Table 4:** Comparison summary of the key advantages and limitations of voltammetric techniques

S. No	Technique	Key advantages	Key limitations
1	Cyclic voltammetry (CV)	Versatility, high efficiency, adaptability, provides mechanistic insight	Matrix interference, less sensitivity
2	Linear sweep voltammetry (LSV)	Simple	Limited dynamic range, poor sensitivity
3	Differential pulse voltammetry (DPV)	Easy applicability, minimum background current, strong sensitivity	Time consuming
4	Square wave voltammetry (SWV)	Excellent sensitivity, fast, electrode kinetic study	Complex data analysis, parameter optimization

Through the comparison of these voltammetric techniques, researchers gain insights into the specific strengths and limitations of each method. Cyclic voltammetry is particularly recommended for comprehensive qualitative analysis and mechanistic investigations, while linear sweep voltammetry serves as a valuable tool for fundamental electrochemical assessments. DPV and SWV are more appropriate for analyses requiring high sensitivity. Additionally, researchers can validate their experimental findings in electrochemical studies by juxtaposing them with theoretical outcomes derived from computational analyses.

## 8. Density functional theory study of anticancer and antibiotic drugs

**Fig. 9:**
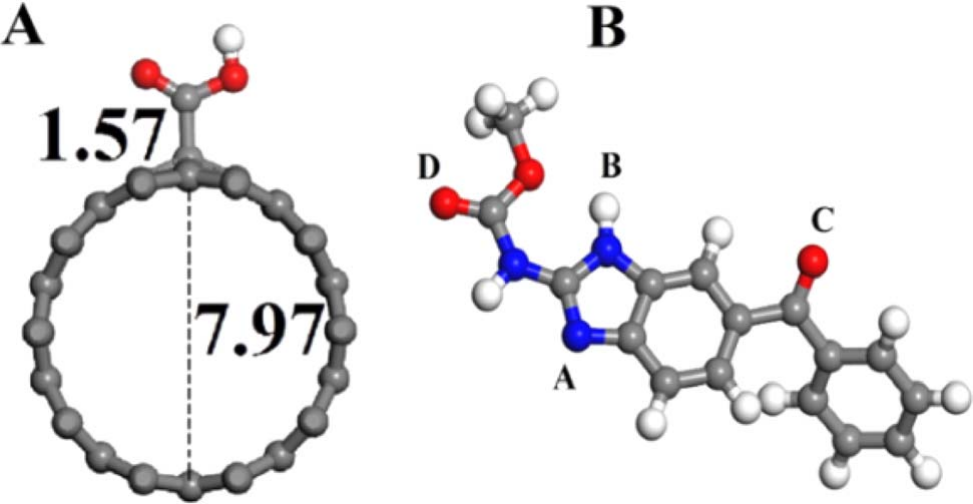
(A) Front image of supercell optimized with (10–0) functionalized carbon nanotubes. (B) Different optimized configuration (A, B, C, D) of MBZ while atoms C, H, O and N are represented by blue, grey, red and white balls.^94^ Reproduced with permission from ref. 94. Copyright (2016) *Mater. Sci. Eng.*

**Fig. 10:**
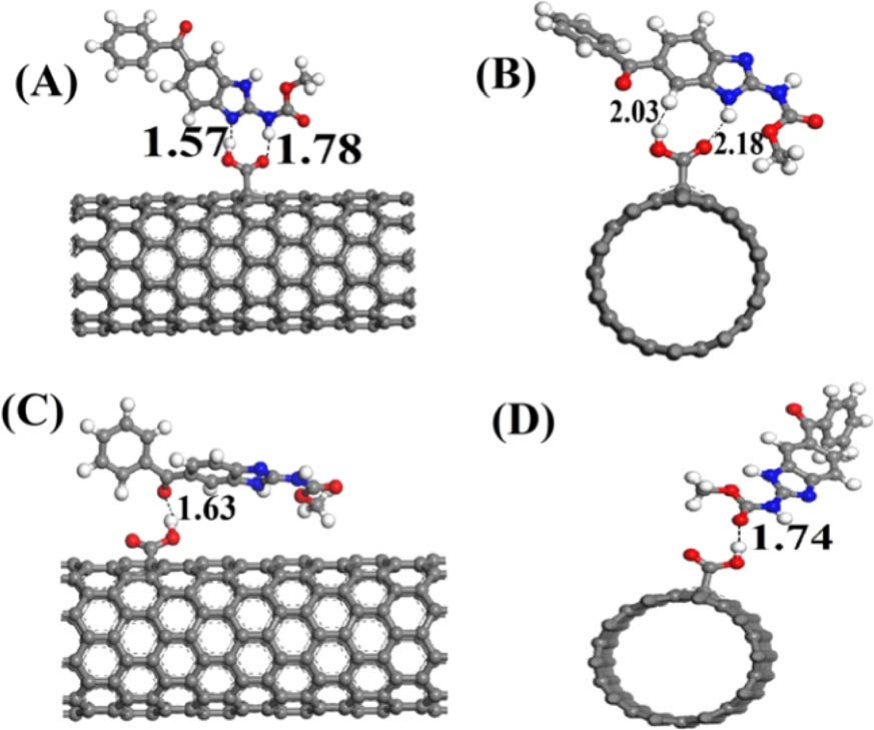
The optimized configuration of MBZ on functionalized CNTs is represented by (A), (B), (C), (D) and bond distance expressed in angstrom.^94^ Reproduced with permission from ref. 94. Copyright (2016) *Mater. Sci. Eng.*

Density functional theory (DFT) serves as a valuable tool for examining and predicting the redox potential, reactivity of pharmaceutical compounds, and their electronic structure.^166^ By focusing on electron density and its correlation with external potential, DFT facilitates the estimation of various characteristics, including molecular geometry, energy levels, and reaction parameters.^167^ Despite its limitations, DFT remains instrumental in understanding and forecasting electron behavior in atoms and molecules.^168^ This review extensively investigates the oxidation mechanism of MBZ through voltammetric methods, while also employing DFT alongside experimental data to elucidate the adsorption process of MBZ on the modified electrode surface. The findings obtained were intended to clarify the interaction mechanism of MBZ with the (10−0) carboxylic acid functionalized CNT-modified electrode surface, as well as to detail the electrical and structural properties of CNT-based sensors affected by the adsorption process. The optimized structure of CNTs is illustrated in Fig. 9A, while Fig. 9B depicts the functionalization of CNTs with various groups, including amine, imine, ester, and carbonyl in configurations A, B, C, and D. The results indicated that configuration A exhibited a higher adsorption energy (*E*_ads_ = −0.351 eV), a shorter distance, and an increased charge transfer between the drug MBZ and the functionalized CNTs, confirming effective adsorption. The bond distances for the different configurations are presented in Fig. 10. Furthermore, functionalized CNTs were identified as effective electrode modifiers, characterized by a lower and negative adsorption energy value (*E*_ads_ = −0.770 eV) and minimal impact on the electronic structure of fCNT, thereby enabling a straightforward, rapid, and precise method for the careful assessment of drugs.^94^

Xiao and colleagues explored the sensing mechanism for detecting fluoroquinolone antibiotics, specifically ofloxacin and ciprofloxacin, utilizing DFT simulations to calculate the LUMO and HOMO energy levels *via* B3LYP/6-31G*. This research supports the photoinduced electron transfer theory and provides significant insights into the development of fluorescent sensing materials for antibiotics applicable in various fields.^169^ By integrating computational studies with electrochemical sensing, a deeper understanding of drug interactions and properties is achieved, facilitating advancements in sensor design, pharmaceutical formulations, and the assessment of pharmaceutical behavior across different matrices, thereby enhancing the accuracy and reliability of drug analysis.^170^

## 9. Challenges and future perspectives

Voltammetric techniques are particularly effective for investigating the redox properties of active pharmaceutical ingredients, offering insights into drug mechanisms and *in vivo* redox processes. However, these methods face several challenges, notably the need for the development of selective, reliable, sensitive, and reproducible sensors.^171^ A major issue is the overpotential required to initiate electron transfer in redox reactions, which hampers sensitivity and practical application in real-world scenarios. Additionally, the manual processing of samples involves multiple steps that can introduce systematic errors and prolong the electrochemical analysis.^172^ Moreover, interference from other biomolecules or metabolites may result in false positives or negatives due to structural similarities, complicating the analysis further. While the integration of nanotechnology is progressing rapidly, it remains in its early stages, particularly in the medical field, and its practical applications in real-world settings have yet to be fully realized.

Future developments present significant opportunities for the production of electrochemical sensing devices tailored for advanced applications in pharmaceuticals, biology, and clinical diagnostics. It is essential to improve the accuracy and reliability of these methods for monitoring pharmaceuticals in both environmental and clinical settings by refining techniques and enhancing the synthesis of nanomaterials used as sensing platforms. Ensuring sensor reproducibility is crucial for commercialization. Additionally, conducting *in vivo* measurements will support research that mimics natural physiological conditions, while prioritizing miniaturization techniques for electrodes is vital to reduce potential harm to subjects. This includes the development of wearable and implantable sensors. Given the critical role of nanomaterial-based electrochemical sensors, they are anticipated to effectively analyze real samples without the need for complex preparation. Moreover, the application of molecularly imprinted polymers (MIPs) in chemical and biosensors represents a promising research avenue. There is also considerable interest in multi-target sensors designed to detect emerging environmental contaminants, enabling simultaneous multiplex sensing of analytes, thereby enhancing the efficiency and effectiveness of the technique. Efforts must focus on enhancing electrode modification strategies to guarantee the long-term stability of electrodes. Advances in sensor technology have facilitated the use of sustainable materials, which can reduce costs and enhance affordability, although initial expenses may still be high, particularly in resource-limited environments. Therefore, there is a pressing need for cost-effective electrochemical sensors to ensure accessibility for the general public.^173^ It is expected that future developments will increasingly emphasize the use of biodegradable materials in sensors and their incorporation into microfluidic devices, paving the way for environmentally friendly and highly efficient automated detection systems.

## 10. Conclusion

Undoubtedly, the production and application of anticancer and antibiotic medications have improved health status and wellbeing. However, protection from the toxic effects of drugs and understanding their mechanism of action demands further research. This document on the detection of pharmaceutical residues brings attention to the importance of nanomaterials based electrochemical sensors for the protection of human health. This work highlights voltammetry as a highly efficient tool for examining the redox characteristics of drugs and their low-level detection. The versatile, environmentally benign, and sustainable nature of the voltammetric technique makes it a valuable tool for monitoring and analysis. This review presents the redox mechanisms of anticancer and antibiotics based on results obtained from electroanalytical techniques that give awareness about the metabolic fate of these drugs. Nanomaterials based electrochemical sensors play a fundamental role in revealing the existence of pharmaceutical residues that pose a risk to ecosystem while the difficulties being encountered in the use and selection of electrode modifiers for enhancing sensing events suggest this field as a hot area of future research. DFT studies suggest that integration of theoretical chemistry and electrochemical sensing can provide valuable mechanistic insights about electron transfer events between the electrode and redox responsive drug residue. This review presents the versatility of the electrochemical techniques for drug detection and analysis and encourages further research and innovation in environmental and health sectors. It can support decision-making for reducing the impact of hazardous residues on human health and water dwelling organisms by giving precise and timely data on pharmaceutical effluents. Future research should focus on electrode modifiers that enhance sensitivity characteristics, probing novel interfaces for efficient detection, and improving data analysis methods to overcome onsite detection challenges.

## Data availability

The authors declare that the data are available in this manuscript in the form of tables and figures.

## Conflicts of interest

The authors declare no conflict of interest regarding the publication of this manuscript.
